# Biosynthesis of polyamine-polyphosphate granules for colitis alleviation

**DOI:** 10.1016/j.isci.2026.114679

**Published:** 2026-01-12

**Authors:** Zihao Fan, Xuena Zhang, Xiaotong Ge, Lei Zhu, Minsheng Zhu, Liuyan Yang

**Affiliations:** 1 Model Animal Research Center, Medical School, Nanjing University, Nanjing, Jiangsu Province 210031, China; 2 State Key Laboratory of Water Pollution Control and Green Resource Recycling, School of Environment, Nanjing University, Nanjing, Jiangsu Province 210023, China; 3 Department of Gastroenterology and Central Laboratory of Nanjing Drum Tower Hospital Group Suqian Hospital, Suqian, Jiangsu Province 223800, China; 4 Wuxi Fisheries College, Nanjing Agricultural University, Wuxi, Jiangsu Province 214081, China

**Keywords:** Biosynthesis, Biological sciences, Biomaterials

## Abstract

Natural polyamines, such as spermidine and spermine, exert strong anti-inflammatory effects, but their therapeutic benefits are limited by rapid absorption in the small intestine, resulting in low colonic availability. In this study, engineered *Citrobacter freundii* overexpressing *ppk1* (CPP) could significantly enhance both intracellular polyamine synthesis and polyphosphates (PolyP) accumulation. Under a two-step cultivation process, the engineered strain produced substantial amounts of polyamines, including spermidine (145.14 ± 5.11 mg/g) and spermine (175.31 ± 4.2 mg/g). CPP responds to environmental stress by importing large amounts of phosphate for PolyP synthesis. This process further promotes polyamine production, which binds to PolyP to neutralize intracellular charge. The resulting polyamine-polyphosphate granules (PPGs) enhance both binding stability and slow-release properties *in vivo*. Notably, the oral administration of PPGs increased colonic polyamine levels, alleviated DSS-induced colitis, reduced inflammatory cytokines, and restored gut microbiota balance. Overall, biosynthesized PPGs represent a highly promising material for mitigating colitis.

## Highlights

• Engineered *C. freundii* accumulates abundant polyamines and PolyP under two-step cultivation
• Phosphate uptake drives intracellular assembly of polyamines and PolyP into PPGs
• PolyP in PPGs enables the slow release of polyamines and increases colonic polyamine levels
• Oral PPGs alleviate colitis by restoring immune function and gut microbiota homeostasis

## Graphical abstract

**Figure:**
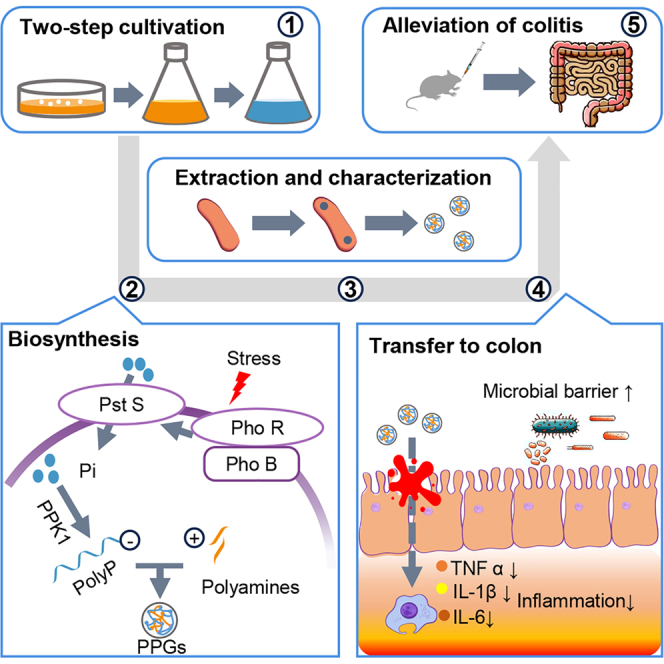

## Introduction

Polyamines, such as spermidine and spermine, are small, positively charged aliphatic compounds widely found in living organisms.^1^^,^^2^ They play essential roles in stabilizing nucleic acid structures, regulating gene expression, and maintaining key cellular processes, including proliferation, differentiation, redox balance, and apoptosis.^3^ In the intestine, polyamine levels are tightly regulated and primarily sourced from the diet and gut microbiota.^4^^,^^5^ Beyond supporting epithelial turnover, polyamines are critical for maintaining intestinal barrier integrity and immune homeostasis. Consequently, their dysregulation has been closely associated with inflammatory bowel disease (IBD).^6^^,^^7^ Mechanistically, natural polyamines alleviate colitis by inhibiting the differentiation of pro-inflammatory macrophages while promoting the accumulation of anti-inflammatory macrophages.^8^ Nonetheless, oral administration of polyamines shows limited efficacy in treating colitis, as they are rapidly absorbed in the small intestine and enter systemic circulation, resulting in inadequate delivery to the colon. Microbial preparations have been considered as a promising approach to elevate luminal polyamine levels. For instance, consumption of yogurt containing the bifidobacterial strain LKM512 combined with free arginine has been shown to elevate intestinal putrescine levels, extend lifespan, and improve multiple physiological and cellular markers of aging.^9^^,^^10^ Similarly, colonization of germ-free mice with polyamine-producing *Escherichia coli* WT but not the polyamine-deficient strain *Escherichia coli* SK930 promoted recovery from colitis.^11^ However, under colitis conditions, the administration of live bacteria is often undesirable due to the increased risk of infection. These limitations highlight the need for safer alternatives, such as the use of bacterially derived polyamines in the form of postbiotics for targeted colonic delivery.^12^

Similar to polyamines, polyphosphate (PolyP) is a long-chain anionic polymer synthesized through microbial metabolism. Intracellular PolyP acts not only as a reservoir of inorganic phosphate but also plays roles in energy metabolism, stress adaptation, and metal ion homeostasis. Depending on environmental conditions, PolyP can adopt various physical states such as soluble molecules, nanoparticles, or coacervates, which are known as metachromatic granules. Nevertheless, the intracellular interaction between polyamines and PolyP remains poorly understood. As a drug delivery platform, PolyP nanoparticles can be triggered by pH variations or protein interactions, allowing precise spatiotemporal control over drug release.^13^ A typical application involves the encapsulation of dexamethasone and vitamin C into a core-shell delivery system, enabling synergistic dual-drug release to enhance bone tissue regeneration.^14^ Growing evidence also underscores the therapeutic promise of biologically derived PolyP/polyamine complexes as postbiotic agents for managing colitis.^15^^,^^16^ These microbial derivatives exhibit improved safety profiles compared to live probiotic formulations. For instance, PolyP nanoparticles from *Synechococcus* sp. PCC 7002 has been shown to strengthen the intestinal barrier and alleviate colitis symptoms.^17^ It is hypothesized that combining cationic polyamines with anionic PolyP to form nanoparticles or granules could enhance the stability and enable sustained-release of the complexes, while also significantly improving spermidine bioavailability in the colon.

Moreover, microbial metabolites have shown great promise as therapeutic agents, with additional advantages for large-scale manufacturing. To achieve efficient biosynthesis of these compounds, a two-step cultivation strategy is widely adopted, facilitating both microbial activation and improved metabolite production during fermentation. This approach generally consists of a pre-cultivation stage for cell activation or enrichment, followed by a second stage optimized for selective growth or metabolite synthesis. Such a strategy not only increases product yield and enables the cultivation of challenging strains but also offers enhanced process control. For instance, it has been successfully implemented in the production of poly-hydroxybutyrate using *Methylocystis* sp.,^18^ succinic acid with engineered *Mannheimia succiniciproducens*,^19^ and vitamin B6 in engineered *E. coli*.^20^ In this study, we bridged metabolic engineering with medical application using a two-step biosynthesis strategy in engineered *Citrobacter freundii* overexpressing *ppk1* (hereafter CPP). The process involved first culturing CPP cells in Luria-Bertani (LB) medium for biomass expansion, followed by transfer to a defined synthetic medium (SM). This strategy facilitated the substantial accumulation of PolyP and polyamines, which subsequently assembled into insoluble polyamine-polyphosphate granules (PPGs). Orally administered PPGs elevated colonic polyamine levels and alleviated dextran sulfate sodium (DSS)-induced colitis in mice, underscoring their promise as bio-derived carriers for targeted intestinal therapy.

## Results

### Two-step cultivation facilitates the formation of granules in CPP

**Figure 1:**
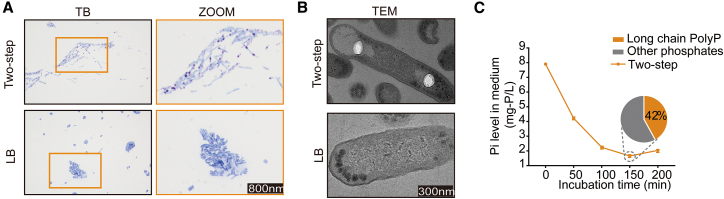
Two-step cultivation of engineered *C. freundii* promotes PolyP accumulation and granule formation (A and B) Morphological analysis of intracellular granules in engineered *C. freundii* (CPP) cultured under Two-step and Luria-Bertani (LB) medium conditions. Representative images from optical microscopy and transmission electron microscopy (TEM) are shown. Scale bars: 800 nm (optical) and 300 nm (TEM). (C) Phosphate uptake by CPP shows enhanced PolyP synthesis under Two-step cultivation (*n* = 3).

After two-step cultivation, the granules became physically segregated from the cytoplasmic compartment and exhibited a distinct dumbbell-shaped morphology. Upon staining with toluidine blue, the granules showed characteristic purple metachromasia, indicative of a high PolyP content (Figures 1A and 1B). Approximately 42% of the phosphate absorbed from the medium was allocated to the formation of these intracellular granules (Figure 1C).

### The extracted granules contain long-chain PolyP and polyamines

**Figure 2:**
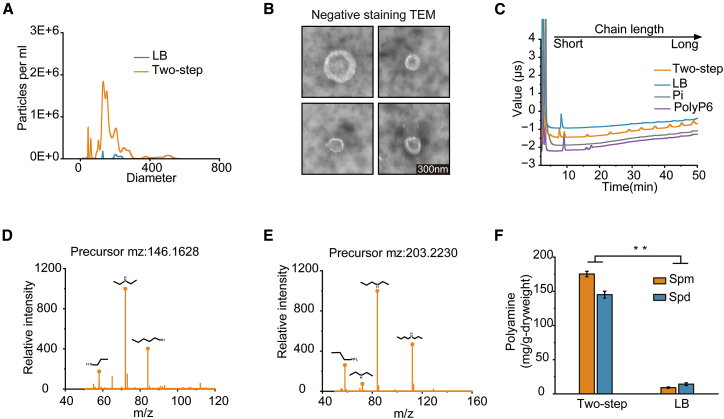
Granules in CPP are enriched with long-chain PolyP and polyamines (A) Granule size distribution profiles determined by nanoparticle tracking analysis (NTA). (B) Negative-stain TEM images of the extracted granules. Scale bars: 300 nm. (C) Chain length distribution of PolyP within granules analyzed by ion chromatography (IC). (D and E) MS/MS spectra of spermidine and spermine extracted from granules (Level 2 confidence). (F) Quantification of Intracellular spermine and spermidine during granule formation in CPP (Level 1 confidence). All quantitative data represent mean ± standard deviation (*n* = 3). Significance was determined by paired two-tailed *t* test (∗*p* < 0.05, ∗∗*p* < 0.01).

Analysis of extracts fractionated by Sephadex G-200 confirmed the presence of phosphate-rich granules (Figure S1). The extracted granules ranged in size from 100 to 200 nm (Figures 2A and 2B). Ion chromatography (IC) using inorganic phosphate (Pi) and hexametaphosphate (PolyP_6_) as standards showed that extracts from two-step cultivated CPP contained abundant long-chain PolyP, whereas no corresponding signal was detected in extracts from LB-grown cultures, indicating the PolyP enrichment in the granules (Figure 2C). In ESI^+^ mode, MS/MS analysis using the MS-DIAL database identified spermidine (m/z 146.1628) and spermine (m/z 203.2230) as the major cationic components of the extracts (Figures 2D and 2E). Quantitative analysis revealed significantly higher levels of spermidine (145.14 ± 5.11 mg/g) and spermine (175.31 ± 4.2 mg/g) in CPP cultured in SM medium compared to LB-grown cultures (14.16 ± 2.31 mg/g and 9.13 ± 2.5 mg/g, respectively) (Figure 2F). These results indicate that two-step cultivation greatly enhances the production of both spermidine and spermine in CPP.

### Phosphate uptake promotes the upregulation of polyamine synthesis in CPP

**Figure 3:**
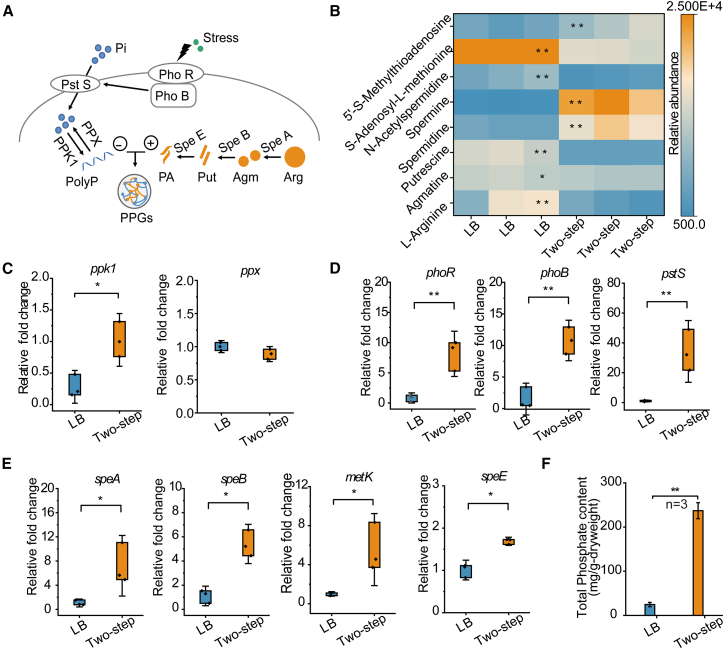
Phosphate uptake promotes granule formation (A) Schematic diagram of the PolyP and polyamine metabolic pathways in CPP. Components include orthophosphate (Pi), arginine (Arg), agmatine (Agm), putrescine (Put), spermidine (Spd), and spermine (Spm). (B) Relative abundance of intermediate metabolites involved in PolyP and polyamine metabolism in CPP grown in LB and Two-step. Blue indicates low relative abundance; orange indicates high relative abundance (significant differences observed). (C–E) mRNA expression profiles of CPP cultured in LB (blue) and two-step (orange) groups. Genes related to polyphosphate metabolism include *ppk1* (polyP synthesis), *ppx* (polyP degradation), *phoB-phoR* (two-component regulatory system), and pstS (phosphate transporter). Genes related to polyamine biosynthesis include *speA* (arginine decarboxylase), *speB* (agmatine ureohydrolase), and *speE* (spermidine synthase), which convert arginine into spermidine and spermine. *metK* encodes S-adenosylmethionine synthetase, providing the aminopropyl donor for spermidine synthesis. (F) The total phosphate content of extracted PPGs. Data are presented as mean ± standard deviation (*n* = 3). ∗*p* < 0.05, ∗∗*p* < 0.01.

To investigate the accumulation patterns of PolyP and polyamines in CPP, the relevant metabolites and gene expression profiles were examined (Figure 3A). The resulting metabolomic data revealed a distinct signature: depletion of pathway precursors (arginine and putrescine) and metabolic intermediates (S-adenosylmethionine and N-acetylspermidine), coupled with the accumulation of end-products (spermidine and spermine) and the byproduct 5′-methylthioadenosine (Figure 3B). The metabolite profilesuggested that PolyP accumulation in CPP was directly linked to the formation of PPGs. Furthermore, untargeted metabolomic analysis focusing on polyamine-related metabolites demonstrated clear separation between the LB and two-step groups. Specifically, under two-step cultivation, relative levels of arginine (Arg), putrescine (Put), S-adenosylmethionine, and N-acetylspermidine were reduced, while those of 5′-methylthioadenosine, spermidine, and spermine were significantly elevated. Under two-step cultivation, the expression levels of genes encoding the phosphate regulatory system increased significantly compared to LB culture: the phosphate two-component regulatory protein gene (*phoB*) and the sensor kinase gene (*phoR*) were upregulated by 8.3-fold and 7.5-fold, respectively (Figures 3C and 3D). Consistent with this activation, the phosphate-binding protein gene (*pstS*) exhibited a 38.5-fold upregulation, indicating a substantial enhancement in intracellular phosphate availability. Meanwhile, the expression of *ppk1*, which catalyzes the conversion of Pi into PolyP, was also highly elevated. By contrast, the expression of the PolyP degradation-related gene *ppx* remained unchanged. ELISA further confirmed that the two-step cultivation method significantly increased the intracellular abundance of polyphosphate kinase (PPK). Since the CPP genome lacks a *ppk2* homolog, this elevated PPK signal is primarily attributable to PPK1. Consistent with the heightened PPK1 level, a pronounced decrease in intracellular ATP was observed during the two-step process, indicating strong activation of PPK1 to consume ATP, thereby driving PolyP synthesis (Figures S2A and S2B). In the polyamine synthesis pathway, genes encoding arginine decarboxylase (*speA*), agmatinase (*speB*), and spermidine synthase (*speE*) were significantly upregulated. This was accompanied by the increased expression of *metK*, which encodes S-adenosylmethionine synthetase (Figure 3E). Correlated with these genetic changes, quantitative analysis revealed that the total phosphate content in the two-step extract reached 12 mg/L (Figure 3F), indicating significantly higher intracellular phosphate levels in CPP under two-step cultivation compared to the LB method. Furthermore, at this stage, the polyamine level in the extract reached 704.9 mg/L, corresponding to a total polyamine concentration of 3 mM (calculated as spermidine equivalents).

### PolyP binds with polyamines to form granules

**Figure 4:**
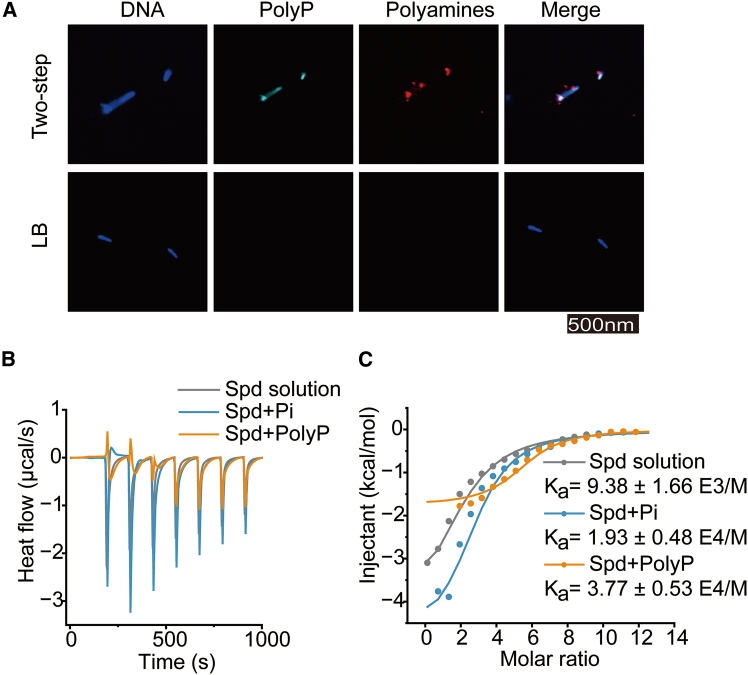
Polyamines interact with PolyP to form stable granules (A) Spatial colocalization of PolyP and polyamines within granules visualized by confocal microscopy. Fluorescence channels: DAPI-DNA (403/425–475 nm), DAPI-PolyP (403/552–617 nm), Polyamine-Red (560/585 nm), and merged composite. Scale bars: 500 nm. (B) Isothermal titration calorimetry (ITC) thermograms depicts the titration of Pi/PolyP into Spd solution. (C) Binding isotherms fitted to a one-site model for PA solution, Spd + Pi, and Spd + PolyP.

Critical evidence supporting PPGs' formation was obtained from dual fluorescence staining. The co-localization of DAPI and polyamine-RED signals within metachromatic granules conclusively demonstrated their dual composition of polyamines and PolyP (Figure 4A). In CPP under two-step cultivation, polyamines and PolyP co-assembled following an identical pattern, segregated into the same granular structures. To quantitatively evaluate the binding propensity between PolyP and polyamines, we performed isothermal titration calorimetry (ITC) to examine the interactions of PolyP_6_ or Pi with spermidine *in vitro*. ITC measurements revealed that the number of binding sites (n) in the Spd + Pi system (*n* = 2.79) was comparable to that of free spermidine in solution (*n* = 2.14). In stark contrast, the Spd + PolyP system exhibited a substantially higher binding site occupancy (*n* = 5.45). Notably, the binding affinities ranked in the order: Spd + PolyP > Spd + Pi > Spd alone, with association constants (K_a_) of 3.77 × 10^4^, 1.93 × 10^4^, and 9.38 × 10^3^ M^−1^, respectively (Figures 4B and 4C). Consistent with this trend, the ΔG values decreased progressively from −5.42 kcal mol^−1^ (Spd) to −5.85 kcal mol^−1^ (Spd + Pi) and −6.24 kcal mol^−1^ (Spd + PolyP) (Table S1), indicating that the polyamine-PolyP complex possesses the highest stability and the strongest binding interaction under the tested conditions.

### Oral administration of polyphosphate granules alleviates colitis

**Figure 5:**
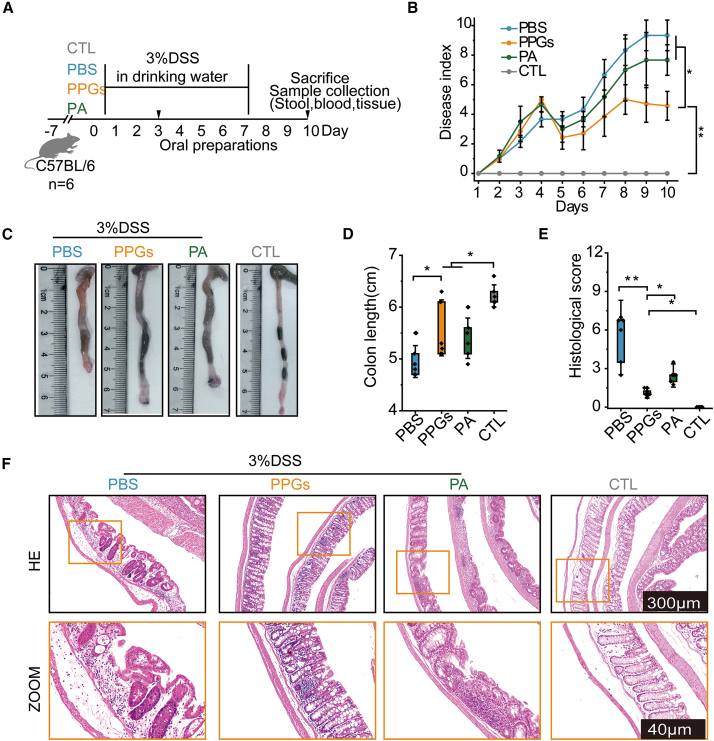
Oral administration of PPGs alleviates DSS-induced colitis in mice (A) Experimental colitis model with oral treatments reveals the therapeutic effect of PPGs. (B) Clinical disease progression monitored by disease Index (DAI) scores across different treatment groups with PBS, PPG, and PA. (C and D) Representative image of the colons and the values of colon length. (E) Histological scores of the colon in treatment groups with PBS, PPG, and PA. (F) Hematoxylin and eosin-stained images of representative histopathologic changes. Data are presented as mean ± standard deviation (*n* = 6). Statistical significance: ∗*p* < 0.05, ∗∗*p* < 0.01 by one-way ANOVA.

These results establish PPGs as stable supramolecular assemblies. Prior to evaluating their therapeutic potential, we confirmed the safety profile of PPGs. IC confirmed that PolyP was exclusively detected in mice administered with PPGs (Figure S3). Ten days of continuous oral administration were well-tolerated, causing no changes in body weight, colonic histopathology (Figure S4), or abundance of pathogenic bacteria in healthy mice (Figure S5). We then evaluated the therapeutic efficacy of PPGs in a DSS-induced colitis mouse model, randomizing animals into four groups (*n* = 6, Figure 5A). From day 6 onward, PPG-treated mice exhibited strikingly lower DAI scores and minimal weight loss (Figure S6), in contrast to PA and PBS groups, which showed similar disease progression (Figure 5B). PPGs administration significantly mitigated DSS-induced colon shortening (5.6 ± 0.5 cm vs. 5.0 ± 0.3 cm in PBS controls), while PA offered no protective effect (Figures 5C and 5D). Histopathological analysis further demonstrated substantial improvements in PPG-treated mice, characterized by preserved epithelial integrity and diminished inflammatory cell infiltration, resulting in significantly lower histological scores compared to both control groups (Figures 5E and 5F).

### Oral polyphosphate granules elevate colonic spermidine levels and suppress pro-inflammatory cytokines

**Figure 6:**
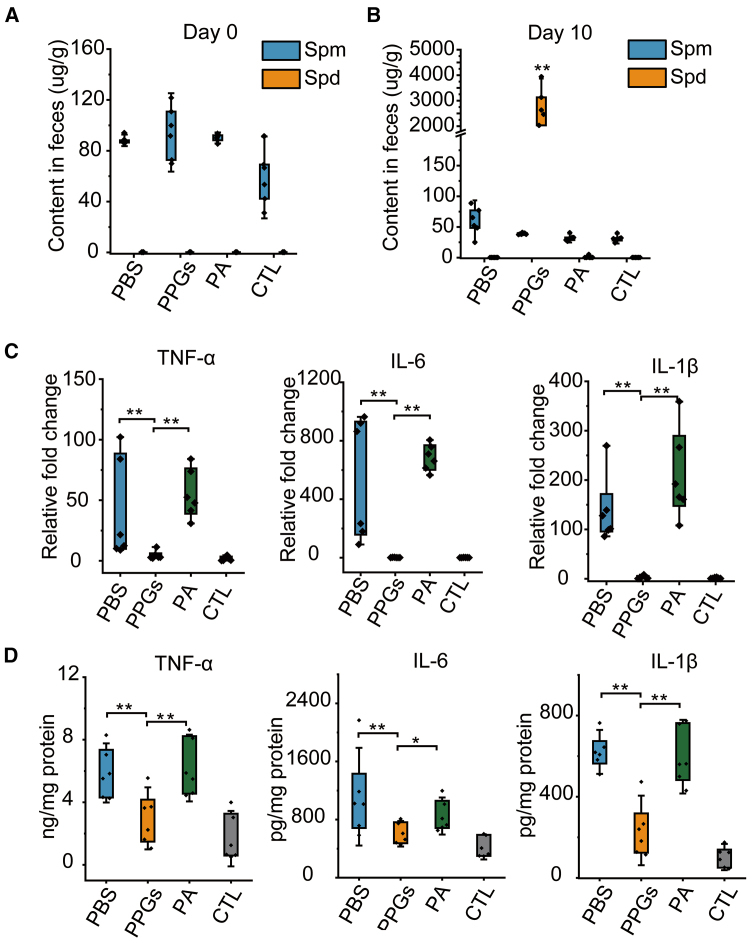
Oral PPGs elevate colonic spermidine and suppress pro-inflammatory cytokines. Polyamine concentrations were measured (A) before and after (B) PPGs administration. Data are expressed as mean ± SD. Statistical significance: ∗*p* < 0.05, ∗∗*p* < 0.01 by one-way ANOVA. (C and D) Inflammatory cytokine levels in colonic tissue. mRNA and protein expression levels and of tumor necrosis factor-α (TNF-α), interleukin-6 (IL-6), and interleukin-1β (IL-1β) in colon tissues. Data are presented as mean ± standard deviation (*n* = 6). Statistical significance: ∗*p* < 0.05, ∗∗*p* < 0.01 by one-way ANOVA.

To evaluate changes in fecal polyamine levels before and after PPG administration, fecal samples were collected from DSS-induced colitis mice and analyzed via HPLC. Before treatment, the fecal spermine level was 82.9 ± 12.0 μg/g, while spermidine was undetectable (Figure 6A). By the end of the experimental period, fecal spermidine levels in the PPG-treated group increased to 2.6 ± 0.8 mg/g, whereas spermidine remained undetectable in the PA-treated group (Figure 6B). Cytokine analysis demonstrated that PPG-treatment significantly suppressed the expression of key inflammatory mediators (TNF-α, IL-1β, and IL-6), whereas elevated cytokine levels persisted in both the PA- and PBS-treated groups (Figure 6C). When normalized to total protein, cytokine concentrations were quantified as follows: in the PBS group, TNF-α was 5.88 ± 1.57 ng/mg, IL-6 was 1114.96 ± 560.74 pg/mg, and IL-1β was 620.90 ± 83.40 pg/mg. In the PPG-treated group, these levels were markedly reduced to 2.97 ± 1.65 ng/mg for TNF-α, 604.55 ± 146.13 pg/mg for IL-6, and 233.87 ± 131.72 pg/mg for IL-1β. In contrast, cytokine levels in the PA group (TNF-α: 6.19 ± 1.78 ng/mg; IL-6: 849.19 ± 212.68 pg/mg; and IL-1β: 597.48 ± 139.61 pg/mg) were comparable to those in the PBS group. The control (CTL) group exhibited the lowest cytokine concentrations (TNF-α: 1.68 ± 1.48 ng/mg; IL-6: 420.08 ± 140.79 pg/mg; IL-1β: 102.70 ± 49.77 pg/mg) (Figure 6D). Collectively, these findings indicate that PPG exerts a substantially stronger anti-inflammatory effect than an equivalent dose of free polyamines.

### Oral polyphosphate granules restore the gut microbiota in colitis mice

**Figure 7:**
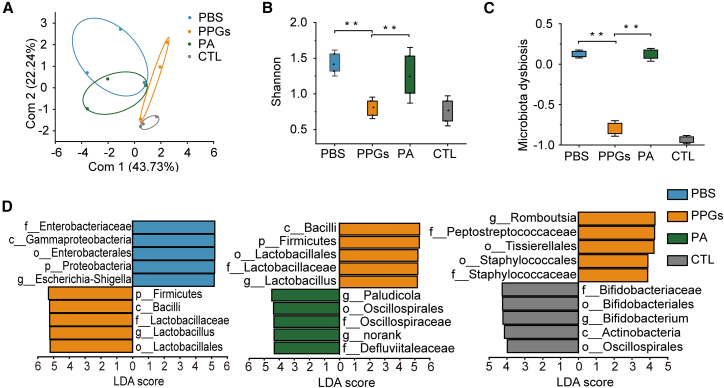
Oral PPGs restore gut microbial community in colitis mice (A) Principal component analysis (PCA) of microbial β-diversity based on unweighted UniFrac distances, illustrating compositional differences among groups. (B) Alpha diversity assessed using the Shannon index, reflecting species richness and evenness. (C) Microbiota dysbiosis index, quantifying deviations from a healthy microbial profile. (D) LDA scores showing taxa with significantly different abundances (left to right: PPGs vs. PBS, PPGs vs. PA, and PPGs vs. CTL). Group differences were evaluated using the Wilcoxon nonparametric test (*n* = 3, ∗*p* < 0.05, ∗∗*p* < 0.01).

To assess the impact of DSS-induced colitis on gut microbiota composition, we performed 16S rRNA high-throughput sequencing on murine fecal samples. Principal component analysis (PCA) of β-diversity, based on unweighted UniFrac distances, revealed distinct clustering of microbial communities among the four experimental groups (PBS, PA, PPG, and CTL) (Figure 7A). The PA group exhibited a microbiota profile closely resembling the PBS group, suggesting that the oral administration of free polyamine failed to alleviate DSS-induced dysbiosis. In contrast, the PPG group displayed a profile intermediate between the PBS and CTL groups, indicating a partial restoration toward a healthy microbial state. These findings were corroborated by α-diversity metrics (Shannon and dysbiosis indices). Both PBS- and PA-treated mice showed significantly reduced microbial diversity after DSS challenge. Notably, the PPG maintained diversity levels comparable to the healthy controls group (Figures 7B and 7C), collectively demonstrating that PPG treatment promotes the beneficial modulation of gut microbial homeostasis during colitis.

At the taxonomic level, the PPG group showed an increased relative abundance of Firmicutes, similar to the CTL group, whereas the PBS- and PA groups were enriched with Bacteroidota and Proteobacteria, suggesting colitis-driven expansion of potentially pathogenic taxa (Figure S4). LEfSe analysis (LDA score >2) identified group-specific microbial biomarkers (Figure 7D). The PBS and PA groups were characterized by inflammation-associated pathogenic taxa, including families such as Enterobacteriaceae and genera such as *Escherichia-Shigella*. Conversely, the PPG group was significantly enriched with beneficial commensals, such as Firmicutes, Bacilli, and Lactobacillales. However, compared with the CTL group, the PPG group still showed elevated abundances of *Romboutsia* and Staphylococcales, reflecting a state of residual dysbiosis (Figures S7A and S7B).

## Discussion

The two-step biosynthesis proposed in this study is fundamentally based on the observation that polyphosphate-accumulating organisms (PAOs), when subjected to environmental stress, tend to utilize PolyP as a carrier. Structurally, PolyP is a linear, branched, or cyclic polymer composed of 3–1000 phosphate units linked by high-energy phosphoanhydride bonds. Its backbone consists of phosphate units interconnected via shared oxygen atoms in a tetrahedral arrangement (P-O-P). Each terminal unit carries two negative charges, while internal units bear one. Given its high negative charge density, excessive intracellular PolyP accumulation imposes a substantial electrostatic burden on the cell, which is typically neutralized through binding with positively charged molecules.^21^ The intracellular charge imbalance suggests a metabolic coupling between polyamine biosynthesis and PolyP accumulation in CPP.

The polyamine production capacity of CPP can be attributed to its *spe*,^22^ a well-characterized genetic cluster in *E. coli* that mediates polyamine synthesis via the arginine-putrescine-spermidine pathway.^23^^,^^24^^,^^25^^,^^26^ Notable successes in genetic modification include the optimization of homospermidine synthase in *Blastochloris viridis*, which yielded a remarkable spermidine production of 725.3–933.5 mg/L through enzyme engineering.^27^ Similarly, *Bacillus amyloliquefaciens* achieved high spermidine production (588.1 mg/L) via a multi-pronged approach involving promoter replacement (native *speB* with P43), knockout of polyamine degradation genes (*yobN* and *bltD*), and medium optimization.^22^ In this study, we enhanced the expression of the native spe gene cluster without genetic modification, resulting in the production of substantial amounts of polyamines (704.9 μg/mL, calculated as spermidine equivalents). Across cultivation stages, CPP adjusts its phosphate metabolism in response to environmental stress. Under nutrition-limited stress in SM, the outer membrane-associated Pho regulatory system becomes activated. In this process, the sensor kinase PhoR phosphorylates the response regulator PhoB,^28^^,^^29^ which then induces the expression of the Pho regulon. This leads to the upregulation of phosphate transporter genes such as *pst*SCAB, facilitating enhanced phosphate uptake from the environment. The increased intracellular phosphate serves as a substrate for PPK1-mediated PolyP synthesis.^30^^,^^31^ The resulting high levels of PolyP interact with intracellular polyamines to form PPGs, which exhibit compartmentalization and a densely packed internal structure. Analogous structures have been reported in prokaryotes, eukaryotes, and even human platelets.^32^^,^^33^^,^^34^ In these systems, PolyP acts as a structural scaffold, while variations in the associated cationic components confer functional diversity.

Beyond their biosynthesis in CPP, understanding the physicochemical basis of PPG is essential for evaluating their potential as delivery systems. In biological systems, polyamines exhibit a strong propensity to form supramolecular assemblies with anionic biomolecules, including nucleotides or other types of PolyP. A key example is nuclear aggregates of polyamines, where electrostatic interactions drive the alternating aggregation of polyamines and nucleotide phosphate groups. Specifically, the amine groups of polyamines bind to phosphate groups, which can further bridge additional polyamine molecules into an extended network.^35^ This process is governed by charge neutralization, which reduces hydration layer thickness and promotes liquid-liquid phase separation, ultimately leading to the formation of dense coacervates. For example, amorphous Ca^2+^–PolyP nanoparticles have been shown to form stable coacervates at physiological pH 7.4, exhibiting high colloidal stability due to their substantial zeta potentials (−34 mV).^36^ Similar to these systems, this study proved that PolyP integrates with polyamines to form PPGs with markedly enhanced thermodynamic stability. ITC results further confirmed this coupling, showing lower ΔG values and stronger binding interactions compared with free polyamines alone.

Based on the ITC data showing strong and thermodynamically favorable interactions between polyamines and PolyP *in vitro*, the CPP system can be utilized as a controlled production chassis for generating these metabolites rather than serving as a live therapeutic. Polyamines themselves are common and well-studied biomolecules, and a variety of purification methods such as ion-exchange chromatography, acid precipitation, ultrafiltration, and reverse-phase HPLC have already been established at both laboratory and industrial scales.^37^ PolyP can also be purified through standard precipitation procedures, selective extraction, and chain-length fractionation.^38^ Therefore, isolating both metabolites from CPP under defined conditions and reconstituting them into synthetic PPGs is technically feasible and scalable. To achieve a GMP-compliant bioproduction process, several additional efforts are necessary. These include strict sterility control and effective removal of endotoxins, establishment of batch-to-batch consistency in PolyP chain length, charge density, and polyamine composition, validation of process reproducibility across different bioreactor scales, and the development of standardized analytical assays to confirm that synthetic PPGs are physicochemically equivalent to their biological counterparts. Meeting these requirements will allow the resulting non-viable and chemically defined PPG particles to comply with modern GMP and ATMP manufacturing standards, providing a practical and scalable route for advancing PPG-based therapies toward clinical application.

In recent years, biomaterial-based therapeutic strategies have advanced rapidly, particularly those employing nano-systems designed to deliver anti-inflammatory or antioxidant agents to inflamed colonic tissue. For example, an EGCG-derived nano-antioxidant platform loaded with curcumin has been shown to effectively alleviate DSS-induced colitis by suppressing oxidative stress, restoring epithelial tight junctions, and modulating inflammatory cytokine production.^39^ In this study, biologically derived PPGs effectively alleviated colitis symptoms by attenuating inflammatory responses, restoring gut microbiota balance, and enhancing local polyamine availability. Further analysis revealed that PPGs not only reduced the levels of pro-inflammatory cytokines but also suppressed the abnormal expansion of opportunistic pathogens such as *E. coli*, *Yersinia*, *Campylobacter*, and *Shigella*, thereby mitigating inflammation-associated dysbiosis.^8^^,^^40^^,^^41^^,^^42^ In IBD treatment, efficient drug delivery and the anti-inflammatory activity of macrophages are considered two key therapeutic goals. The anti-inflammatory effects of polyamines have been well documented.^10^^,^^11^^,^^43^ In this study, the colonic content analysis revealed that the detected polyamine was spermidine, which effectively suppresses the production of pro-inflammatory cytokines by lamina propria immune cells, thereby attenuating the local immune response.^44^ Whereas in PPGs, the abundant long-chain PolyP may facilitate effective transport. Many drugs have exploited such anionic complexes to achieve sustained release and targeted delivery. For instance, polymeric systems such as Eudragit S100 and Eudragit L100 exploit the pH gradient along the gastrointestinal tract to achieve controlled release and colon-targeted delivery.^45^^,^^46^ Compared with these materials, biologically derived PolyP possesses longer chain lengths and more complex self-assembly structures. Studies combining polyamines with PolyP of varying chain lengths have shown that longer chains markedly enhance both the pH stability and thermodynamic stability of the resulting polymers.^47^ Industrial synthesis of long-chain PolyP is challenging because longer chains increase costs, reduce solubility, and complicate processing.^14^^,^^36^ In contrast, biologically synthesized PolyP readily meets these requirements, with chain lengths typically extending to several hundred phosphate units.^48^ Thus, biosynthetic approaches may provide superior delivery performance in practical applications.

In summary, this work demonstrates that PolyP accumulation drives intracellular polyamines enrichment, leading to the formation of PPGs. This metabolic route represents a new approach for generating biologically derived polyamines. Oral administration of PPGs significantly elevated colonic spermidine levels, indicating a slow-release property that ensures sustained local availability. These features highlight the translational potential of PPGs as a bio-derived platform for therapeutic applications in colitis and beyond.

### Limitations of the study

Several limitations should be noted in this study. The molecular architecture of PPGs, including their internal packing features and the chain-length-dependent rules that govern their assembly, remains unclear. In addition, the heterogeneity of biologically synthesized PolyP in chain length and charge density may influence the efficiency of PPGs formation, their stability, and their transport behavior *in vivo*. These factors limit our ability to precisely define the structure-function relationship of PPGs. Future work will focus on achieving the controlled regulation of PolyP chain length in CPP and on characterizing how specific physicochemical parameters shape PPGs assembly and biological performance. Clarifying these mechanisms will deepen our understanding of their supramolecular organization and provide essential guidance for the standardized preparation, quality control, and eventual clinical translation of PPG-based therapeutics.

## Resource availability

### Lead contact

Further information and requests for resources and reagents should be directed to and will be fulfilled by the lead contact, Prof. Liuyan Yang (yangly@nju.edu.cn).

### Materials availability

This study did not generate new unique plasmids, mouse lines, or cell lines.

### Data and code availability

• Data: All raw 16S rRNA sequencing data generated in this study have been deposited in the NCBI Sequence Read Archive (SRA) under the BioProject accession numbers PRJNA1378559 and PRJNA1379157. Raw LC-MS metabolomics data acquired exclusively in positive electrospray ionization mode have been deposited in the MetaboLights repository under accession number MTBLS13621. Additional raw datasets, including ion chromatography profiles, ITC thermograms, and microscopy images, have been deposited in Mendeley Data (Fan, Zihao (2025), “DATA”, Mendeley Data, V1, https://doi.org/10.17632/hyx6sctwm6.1). Accession numbers and related identifiers for these datasets are listed in the key resources table.
• Code: This study did not generate any custom code.
• Other items: Any additional information required to reanalyze the data reported in this article is available from the corresponding author upon reasonable request.

## Acknowledgments

This work was financially supported by the 10.13039/501100001809National Natural Science Foundation of China (No. 41871082 and 42307172).

## Author contributions

L.Y. and M.Z. conceptualized the study. Z.F., X.G., and L.Z. were responsible for methodology. Z.F., X.Z., X.G., and L.Z. carried out the investigations. Z.F. drafted the article, and L.Y. and M.Z. revised it. L.Y. and M.Z. supervised the project.

## Declaration of interests

The authors declare no competing interests.

## STAR★Methods

### Key resources table

**Table:** 

REAGENT or RESOURCE	SOURCE	IDENTIFIER
**Bacterial and virus strains**		

Citrobacter freundii ATCC 8090	American Type Culture Collection	ATCC 8090
*ppk1*-overexpressing *C. freundi*i (CPP)	Yang Lab	This paper

**Chemicals, peptides, and recombinant proteins**		

Spermidine	Macklin	CAS#124-20-9
Spermine	Macklin	CAS#71-44-3
Dextran sulfate sodium	MP Biomedicals	Cat# 160110
Sodium hexametaphosphate	Sigma-Aldrich	CAS#68915-31-1
2-Chloro-L-phenylalanine	Macklin	CAS#103616-89-3
Dansyl chloride	Orileaf	CAS#605-65-2
Polyamine-RED	Dojindo	Cat#211228

**Critical commercial assays**		

Microbial PPK ELISA kit	JSYT	MM-925659O1
ATP assay kit	Jiancheng	A095-1-1
Pierce BCA Protein Assay Kit	Thermo Fisher	23227
Mouse IL-6 OneStep ELISA Kit	STARTER	S0C3019
Mouse TNF-α OneStep ELISA Kit	STARTER	S0C3023
Mouse IL-1β OneStep ELISA Kit	STARTER	S0C3029
FastPure Cell/Tissue Total RNA Isolation Kit	Vazyme	RC101-01
HiScript III 1st Strand cDNA Synthesis Kit (+gDNA wiper)	Vazyme	R312-01
SYBR qPCR Master Mix	Vazyme	Q712-02

**Deposited data**		

Raw and analyzed data	This paper; Mendeley Data	https://data.mendeley.com/datasets/hyx6sctwm6/1
16S sequencing data (safety test)	This paper; NCBI	Table S4; https://www.ncbi.nlm.nih.gov/bioproject/PRJNA1378559
16S sequencing data (colitis)	This paper; NCBI	Table S4; https://www.ncbi.nlm.nih.gov/bioproject/PRJNA1379157
MS/MS data	This paper; MetaboLights	https://www.ebi.ac.uk/metabolights/MTBLS13621

**Experimental models: Organisms/strains**		

C57BL/6 mice	GemPharmatech	E2410080472

**Oligonucleotides**		

qPCR primers used in this study (see Table S1 for full sequences)	Tsingk	Table S1
338F/806R primers for 16S rRNA gene amplification	Majorbio	N/A

**Software and algorithms**		

Origin 2024	OriginLab	https://www.originlab.com/
MS-DIAL	RIKEN	http://prime.psc.riken.jp/
Word	Microsoft	https://www.office.com/
Majorbio Cloud Platform	Majorbio	https://cloud.majorbio.com

**Other**		

Akata pure system	Cytiva	N/A
Sephadex G-200 column	Cytiva	N/A
HPLC-ESI-qTOF 6545	Agilent	N/A
HPLC-2600	Agilent	N/A
ZORBAX Eclipse Plus C18	Agilent	N/A
Sino-chrom ODS-BP C18 column	Elite	N/A
ABI QuantStudio 5 Real-Time PCR System	Thermo fisher	N/A
MicroCal ITC200	Malvern Instruments	N/A
ICS-5000 Ion Chromatography	Thermo fisher	N/A
SpectraMax M3	Molecular Devices	N/A
Olympus FV3000 Confocal Microscope	Olympus	N/A
FEI TECNAI F20 microscope	Thermo Fisher	N/A

### Experimental model and study participant details

#### Microbe strains

The wild-type *Citrobacter freundii* ATCC 8090 strain was obtained from the China Industrial Microbiology Culture Collection (key resources table). The ppk1 gene was amplified from the genomic DNA of *C. freundii* ATCC 8090 and cloned into the medium-copy plasmid pBBR1MCS-2 under the control of a constitutive promoter.^49^ The resulting recombinant plasmid was introduced into the wild-type strain by transformation. Transformants were selected on LB agar plates supplemented with kanamycin, and successful *ppk1* overexpression strains were verified by PCR analysis. The resulting *ppk1*-overexpressing strain was designated CPP.

#### Animals

Male C57BL/6 mice (6–8 weeks old, initial body weight 25–27 g) were obtained from the Nanjing University Model Animal Research Center. All animal experiments were approved by the Institutional Animal Care and Use Committee of the Nanjing University Model Animal Research Center and conducted in accordance with institutional ethical guidelines (ethical approval reference number: AP No.MZ24). Acute colitis was induced by administration of dextran sulfate sodium (key resources table). Mice were maintained in a specific pathogen free room under controlled conditions (22 ± 2 °C; 40–60% relative humidity) on a 12 h light/12 h dark cycle, with standard chow diet and autoclaved water available *ad libitum*.

### Method details

#### Two-step cultivation

The CPP strain was cultured overnight in LB medium at 37.0°C under shaking conditions to ensure uniform growth without PolyP accumulation. Cells were harvested by centrifugation at 8000 rpm for 5 min, washed twice with 20 mM PBS buffer (pH 7.0) to remove residual medium, and then resuspended in the same buffer to obtain PolyP-free cells. To assess the performance of the engineered strain, cells were cultivated in a synthetic medium (SM) devoid of chemical inducers or antibiotics. The SM was prepared with deionized water, contained the following components per liter: 100 mg tryptone, 50 mg NaCl, 226 mg MgSO_4_·7H_2_O, 180 mg NH_4_Cl, and 1 mg yeast extract. Additionally, KH_2_PO_4_·3H_2_O (102 mg/L) was added to maintain a total inorganic phosphorus (Pi) concentration of 20 mg P/L, ensuring non-limiting phosphorus condition unless otherwise stated. PolyP-free cells were inoculated into SM in 1000 mL Erlenmeyer flasks at an initial OD_600_ of 0.15. Cultivation (500 mL) was carried out at 37°C with shaking at 200 rpm. At specified time intervals, 4 mL aliquots were collected for analysis.

#### Purification of PPGs in CPP

The CPP were harvested by centrifugation at 5000 × g for 5 min and resuspended in 5 volumes of HEPES buffer. The cell suspension was then subjected to heat treatment in a boiling water bath for 15 min, followed by rapid cooling in an ice bath. After cooling, the suspension was centrifuged at 6000 × g for 10 min to remove cellular debris. The resulting supernatant, containing bacterial polyphosphate nanoparticles, was loaded onto a Sephadex G-200 column pre-equilibrated with Milli-Q water. Elution was performed with Milli-Q water at a flow rate of 1 mL/min, and the effluent was monitored at 220 nm using an in-line UV detector. The exclusion peak fractions were pooled and concentrated using a 3 kDa ultrafiltration device to obtain the final PPGs stock solution. The phosphate content of the purified PPGs was quantified using the molybdenum blue assay.

#### Metabolites identification

The metabolites of CPP and PPGs were characterized using HPLC-ESI-qTOF. Chromatographic separation was performed on a reverse-phase C18 column with a gradient elution system comprising water, acetonitrile, and methanol, under positive ionization mode.^50^ MS/MS data were acquired in data-dependent acquisition (DDA) mode to enhance structural elucidation. Metabolite identification was conducted using MS-DIAL software, leveraging high-accuracy mass measurements and diagnostic fragmentation patterns with standard mass tolerance settings. Only compounds exhibiting reproducible detection and high spectral matching were considered confidently identified. In accordance with the Metabolomics standards initiative,^24^ these metabolites were classified as Level 2.

#### Extraction and quantification of PolyP

To analyze PolyP, CPP cells were cultured in SM medium for 150 min to facilitate PolyP accumulation. Following incubation, cells were pelleted by centrifugation, washed, and lysed using 2% trichloroacetic acid (TCA) to release intracellular components. After removing the supernatant, PolyP was extracted by sequential treatment with EDTA and phenol/chloroform. To eliminate nucleic acid contamination, the extract was further digested with RNase A and DNase, followed by an additional phenol/chloroform purification step. PolyP was then precipitated with an ethanol–sodium acetate solution, washed with 70% ethanol, air-dried, and resuspended in deionized water. For PolyP quantification, the extracted sample was subjected to acid digestion using concentrated sulfuric acid and hydrochloric acid at high temperature, followed by pH adjusted to 7. Total phosphate content was measured via the phosphor-molybdenum blue method. Briefly, samples were hydrolyzed in 2 N HCl at 95°C for 40 min to convert PolyP to inorganic phosphate (Pi), and the PolyP content (mg-P/L) was subsequently calculated based on the measured Pi.

PolyP chain-length distribution was characterized using an ICS-5000 ion chromatography system. Separation was achieved using a Dionex IonPac AS11 analytical column (2 × 250 mm) coupled with an AG11 guard column (2 × 50 mm). A linear KOH gradient (30–200 mM over 30 min) was applied for elution. A Dionex IonPac AS11 (2 × 250 mm) analytical column and an AG11 (2 × 50 mm) guard column were employed with a 30–200 mM KOH gradient over 30 min. Detection was performed using a Dionex ASRS-ULTRA suppressor in conjunction with a conductivity detector. Longer PolyP chains exhibited later elution times, allowing for chain-length differentiation.^38^

#### Quantitative determination of polyamines

Polyamine extraction was performed as follows: Samples (including bacterial cells and mouse feces) were washed once with ice-cold PBS (4°C) and subsequently extracted with 1 mL of 0.1 M HClO_4_ to release intracellular polyamines. The acidic extract was neutralized to pH 8–8.5 using 2 M NaOH and saturated NaHCO_3_ solution. For derivatization, an equal volume of 10 mg/mL dansyl-chloride solution was added, and the mixture was incubated at 40°C for 30 min in the dark. The reaction was terminated by adding 100 μL of ammonia solution. The derivatized samples were filtered through a 0.22 μm membrane prior to HPLC analysis. Separation was achieved using the HPLC 2600 system with a C18 column. The mobile phase consisted of water:acetonitrile (35:65, v/v) delivered at a flow rate of 1 mL/min. Polyamine derivatives were detected by UV absorption at 254 nm with a total run time of 15 min.^51^ Quantification was performed using external standard curves generated with authentic spermine and spermidine standards.

#### Intracellular polyamine staining

Polyamine-RED was supplemented into fresh culture medium to achieve a final working concentration of 30 μM. Following aspiration of the original medium from bacterial cultures, the samples were washed twice with PBS. Subsequently, the Polyamine-RED-containing medium was added, and the cultures were incubated at 37°C for 1 h. Fluorescence imaging was performed using an Olympus FV3000 confocal laser scanning microscope, with excitation and emission wavelengths set at 560 nm and 585 nm, respectively, for optimal detection of the TAMRA signal.

#### Quantitative RT-PCR

Total RNA was extracted from bacterial cultures, animal tissues, and cultured cells using a commercial kit (key resources table) according to the manufacturer’s instructions. RNA pellets were washed with 75% ethanol, centrifuged, briefly air-dried, and resuspended in DEPC-treated water. RNA concentration and purity were assessed spectrophotometrically. One microgram of RNA was reverse-transcribed into cDNA using a commercial reverse transcription kit (see key resources table). Quantitative PCR was performed on a real-time PCR system with SYBR-based master mix. The 16S rRNA and GAPDH genes served as internal controls for bacterial and eukaryotic samples, respectively. Primer sequences are provided in Table S2.

#### Thermodynamic test

Isothermal titration calorimetry (ITC) measurements were conducted at a constant temperature of 25 °C. The ligand solution (Pi or PolyP) was loaded into the injection syringe, while the sample cell was filled with 3 mM spermidine solution. A reference cell containing distilled water was used to maintain thermal equilibrium. During the experiment, the stirring speed was set to 1,000 rpm, and sequential 1.5 μL aliquots of the ligand solution were injected into the sample cell at 3-s intervals. The resulting thermograms were processed and analyzed using Origin 2024 software. Key thermodynamic parameters, including the binding constant (K_a_), enthalpy change (ΔH), entropy change (ΔS), and binding sites (n), were derived by nonlinear regression fitting to an appropriate binding model.

#### Animal experiments of DSS-induced colitis model

Male C57BL/6 mice (*n* = 6 per group) were acclimated to laboratory conditions for 7 days prior to the experimentation. Acute colitis was induced by administering 3% DSS in drinking water for 7 consecutive days.^52^^,^^53^ Starting on day 3 of DSS administration, mice received a daily oral gavage. The PA group was given 200 μL of a 3 mM mixed polyamine solution (spermidine and spermine at a 1:1 ratio, reported as spermidine-equivalent concentration). The PPGs group was treated with 200 μL of a 3 mM PPG suspension that contained the same total polyamine concentration as the PA solution. And the PBS group received 200 μL of phosphate-buffered saline (PBS). The control group (CTL) received PBS without DSS exposure. All gavage administrations were performed using a feeding needle at a consistent time each day to minimize circadian variability. Following the 7-day DSS challenge period, mice were maintained on normal drinking water for an additional 3 days during the recovery phase.

At the end of the experiment, blood samples were collected in EDTA-coated vacuum tubes and centrifuged at 1500 × g for 15 min at 4°C to isolate plasma. Following blood collection, mice were euthanized, and distal colon tissues (approximately 1 cm proximal to the anus) along with fecal samples were immediately collected. These specimens were rapidly frozen in liquid nitrogen and stored at −80°C for subsequent analysis. The remaining colon segments were carefully prepared using the Swiss roll technique, fixed overnight in 4% paraformaldehyde at 4°C, and then processed through a series of graded ethanol solutions for dehydration. Tissues were subsequently cleared in xylene and embedded in paraffin blocks. Finally, 5 μm-thick sections were prepared using a microtome for histological examination.

The progression of colitis was monitored daily through evaluation of three key clinical parameters: body weight loss, stool consistency, and rectal bleeding. These measurements were systematically combined to calculate a Disease Activity Index (DAI) according to the standardized scoring system detailed in Table S3. This comprehensive index provided a quantitative assessment of colitis severity.^53^

#### ELISA

CPP cultured in LB medium or under the two-step condition were collected and lysed by ice-bath sonication. After centrifugation at 4 °C and 2,500 rpm for 20 min, the supernatant was used to measure PPK protein content. Diluted standards and samples were added to a 96-well plate in triplicate, incubated at 37 °C for 30 min, washed, treated with enzyme conjugate, washed again, and developed with substrate for 10 min in the dark. The absorbance was recorded at 450 nm.

Colonic tissues were homogenized in lysis buffer, and total protein was extracted from the resulting lysates. Protein concentrations were determined using a BCA assay, and all samples were subsequently normalized to a total protein concentration of 50 ng/mL. Equal volumes of the normalized lysates were then added to antibody-precoated ELISA plates and incubated for 1 h at 25 °C. After incubation, the plates were washed according to the manufacturer’s instructions, substrate solution was added, and color was developed for 10 min at room temperature. Absorbance was measured at 450 nm.

#### Histological scoring

Colonic tissue segments (1 cm proximal to the anus) were fixed overnight in 4% paraformaldehyde at 4°C, followed by dehydration through a graded ethanol series, clearing in xylene, and embedding in paraffin. Serial sections (5 μm) were prepared, deparaffinized, rehydrated, and stained with hematoxylin and eosin (H&E) for histological evaluation. A blinded evaluator assessed the tissue sections based on established scoring criteria, evaluating the following parameters: Crypt architecture (0 = normal, 3 = severe distortion), inflammatory cell infiltration (0 = none, 3 = dense infiltration), muscle thickening (0 = normal, 3 = marked thickening), goblet cell loss (0 = absent, 1 = present), and crypt abscess formation (0 = absent, 1 = present). Three randomly selected fields per section were analyzed, and the total histological score was calculated as the sum of all individual parameter scores.^54^

#### DNA extraction from murine fecal samples and 16s rRNA gene sequencing

Fecal microbial DNA was extracted using a commercial kit (key resources table). The V3–V4 region of the bacterial 16S rRNA gene was amplified using the forward primer 338F (5′-ACTCCTACGGGAGGCAGCAG-3′) and the reverse primer 806R (5′-GGACTACHVGGGTWTCTAAT-3′). Sequencing libraries were prepared following standard Illumina protocols for paired-end sequencing (2 × 300 bp). Sequencing was performed on an Illumina MiSeq platform by Majorbio Bio-Pharm Technology Co., Ltd. Raw reads were quality-filtered, merged, and checked for chimeras on the Majorbio Cloud Platform prior to downstream analysis. All raw sequencing data have been deposited in the SRA under the Bio-Project accession number PRJNA1378559 and PRJNA1379157. The accession numbers for all fecal samples are listed in Table S4.

### Quantification and statistical analysis

Raw sequencing data of the 16s rRNA gene were processed and analyzed on the Majorbio Cloud Platform (*n* = 3). Operational Taxonomic Units (OTUs) were clustered at 97% sequence similarity. Microbial diversity within samples was evaluated through alpha diversity indices, including Observed species, Chao1 (richness estimator), and Shannon (diversity index), along with rarefaction curve analysis.

All data are expressed as mean ± standard deviation. Statistical analyses were performed using Origin 2024 software. For group comparisons, either two-way analysis of variance (ANOVA) or unpaired two-tailed Student’s t-tests were employed as appropriate for the experimental design. In specific instances where within-group comparisons were required, paired two-tailed t-tests were utilized. In this study, the sample size will be denoted by n and indicated in both the results section and the figure legends. Statistical significance was defined at two levels: *p* < 0.05 (∗), *p* < 0.01 (∗∗), with non-significant differences denoted as *p* > 0.05.

### Additional resources

This study did not generate additional resources.
