# Supplementary material for: Biosynthesis of polyamine-polyphosphate granules for colitis alleviation
Source: iScience. 2026 Jan 12;29(2):114679. doi: 10.1016/j.isci.2026.114679 (PMC12874136; doi:10.1016/j.isci.2026.114679)
Supplement: Document S1. Figures S1-S7 and Tables S1–S4 [file mmc1.pdf]

**iScience, Volume *29***

## Supplemental information

# Biosynthesis of polyamine-polyphosphate granules for colitis alleviation

**Zihao Fan, Xuena Zhang, Xiaotong Ge, Lei Zhu, Minsheng Zhu, and Liuyan Yang**

**Table S1. Thermodynamic parameters for spermidine binding measured by ITC**

| Titration system | n (sites) | Ka ($M^{-1}$) | Kd (μM) | ΔH (kcal/mol) | ΔS (cal $mol^{-1}$ $K^{-1}$) | ΔG† (kcal /mol) | $\chi^2$/DoF |
|---|---|---|---|---|---|---|---|
| Spd + $H_2O$ | 2.14 ± 0.20 | 9.38 ± 1.66 × $10^3$ | 107 | –4.64 ± 0.54 | 2.62 | –5.42 | 6.86 × $10^3$ |
| Spd + Pi | 2.79 ± 0.22 | 1.93 ± 0.48 × $10^4$ | 51.8 | –4.92 ± 0.54 | 3.10 | –5.85 | 2.21 × $10^4$ |
| Spd + PolyP | 5.45 ± 0.10 | 3.77 ± 0.53 × $10^4$ | 26.5 | –1.76 ± 0.06 | 15.0 | –6.24 | 1.69 × $10^3$ |

**Table S2. qPCR primers list**

| Species | Gene name | Forward | Reverse |
|---|---|---|---|
| C57BL/6 | IL-1β | AAAGATGAAGGGCTGCTTCC | TTCTCCACAGCCACAATGAG |
| C57BL/6 | IL-6 | TAGTCCTTCCTACCCCAATTTCC | TTGGTCCTTAGCCACTCCTTC |
| C57BL/6 | TNF-α | CTGAACTTCGGGGTGATCGG | GGCTTGTCACTCGAATTTTGAGA |
| C57BL/6 | GAPDH(Housekeeping) | TCACCACCATGGAGAAGGC | GCTAAGCAGTTGGTGGTGCA |
| CPP | *speA* | AATCCTACGGCTACAACGG | CACGCTACGGGTCATTCC |
| CPP | *speB* | CAATCCGTCAGGTTTCCA | AGACAGCAGCTTCTCAGCAT |
| CPP | *speE* | TTACGAAGCGTTTGTTGA | CAGACCTTTGCCCTGATA |
| CPP | *metK* | TGGGCGACTGTGGACTG | GCATAGGCTGCGGAACG |
| CPP | *ppk1* | AAGGCCAGCCTTCCGGTATC | GCCTTCCAGGTTCGGGATCA |
| CPP | *ppx* | ACACCGCCCGGGAAATACAT | ATATCGGCGGTGGCTCAACA |
| CPP | *pstS* | CGTTGAGTACGCTTACGCTAA | AAGGTGGTGGAAGTGATTGG |
| CPP | *phoR* | GTGTTGACCACCGAAGAAGGC | ATTGAGCACCAGATTGATAGGC |
| CPP | *phoB* | GTGTTGACCACCGAAGAAGGC | ATTGAGCACCAGATTGATAGGC |
| CPP | 16sRNA(Housekeeping) | ACTCCTACGGGAGGCAGCAG | ATTACCGCGGCTGCTGG |

**Table S3. Disease activity index scoring criteria**

| Score | Weight loss | Stool Consistency | Rectal Bleeding |
|---|---|---|---|
| 0 | 0 % | Normal, well-formed | Negative hemocult |
| 1 | 1-5 % | Soft but still formed | Negative hemocult |
| 2 | 5-10 % | Loose stools | Positive hemocult |
| 3 | 11-18 % | Diarrhea | Mild visible bleeding |
| 4 | ＞18 % | Watery diarrhea | Gross bleeding |

**Table S4. SRA accession numbers for fecal 16S rRNA sequencing samples**

| Accession | Sample group | SPUID |
|---|---|---|
| SAMN53841375 | CTL (healthy mice) | A1 |
| SAMN53841376 | CTL (healthy mice) | A2 |
| SAMN53841377 | CTL (healthy mice) | A3 |
| SAMN53841378 | PPGs (healthy mice) | B1 |
| SAMN53841379 | PPGs (healthy mice) | B2 |
| SAMN53841380 | PPGs (healthy mice) | B3 |
| SAMN53841381 | WT (healthy mice) | C1 |
| SAMN53841382 | WT (healthy mice) | C2 |
| SAMN53841383 | WT (healthy mice) | C3 |
| SAMN53864797 | PBS (mice with colitis) | D1 |
| SAMN53864798 | PBS (mice with colitis) | D2 |
| SAMN53864799 | PBS (mice with colitis) | D3 |
| SAMN53864800 | PPGs (mice with colitis) | E1 |
| SAMN53864801 | PPGs (mice with colitis) | E2 |
| SAMN53864802 | PPGs (mice with colitis) | E3 |
| SAMN53864803 | PA (mice with colitis) | F1 |
| SAMN53864804 | PA (mice with colitis) | F2 |
| SAMN53864805 | PA (mice with colitis) | F3 |
| SAMN53864806 | CTL (healthy mice) | G1 |
| SAMN53864807 | CTL (healthy mice) | G2 |
| SAMN53864808 | CTL (healthy mice) | G3 |

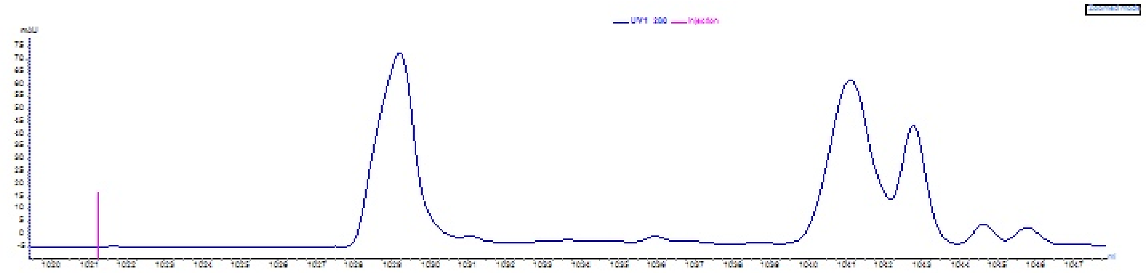


**Figure S1. Representative size exclusion chromatography flow**

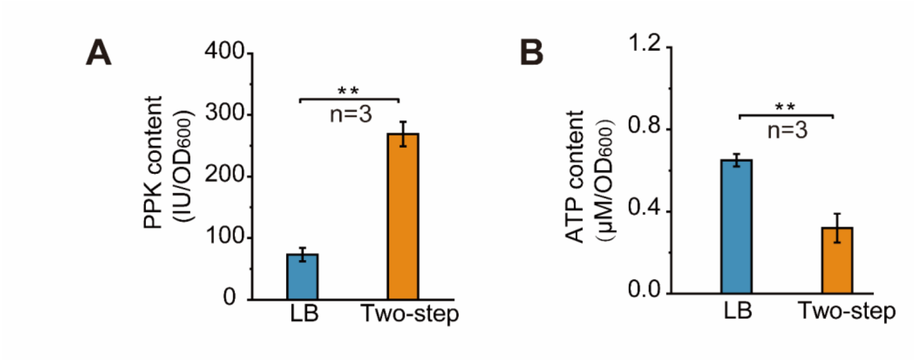


**Figure S2. The intracellular levels of polyphosphate kinase (PPK)** (A) **and ATP** (B) **in CPP under different cultivation conditions**

Data are presented as mean ± standard deviation (n=3). *P < 0.05, **P < 0.01.

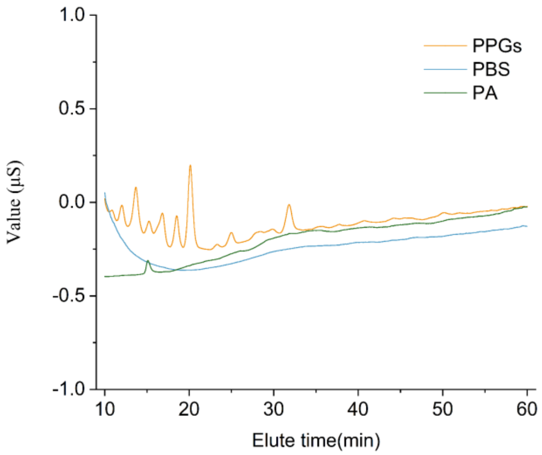


**Figure S3. IC analysis of PolyP in the oral solution**

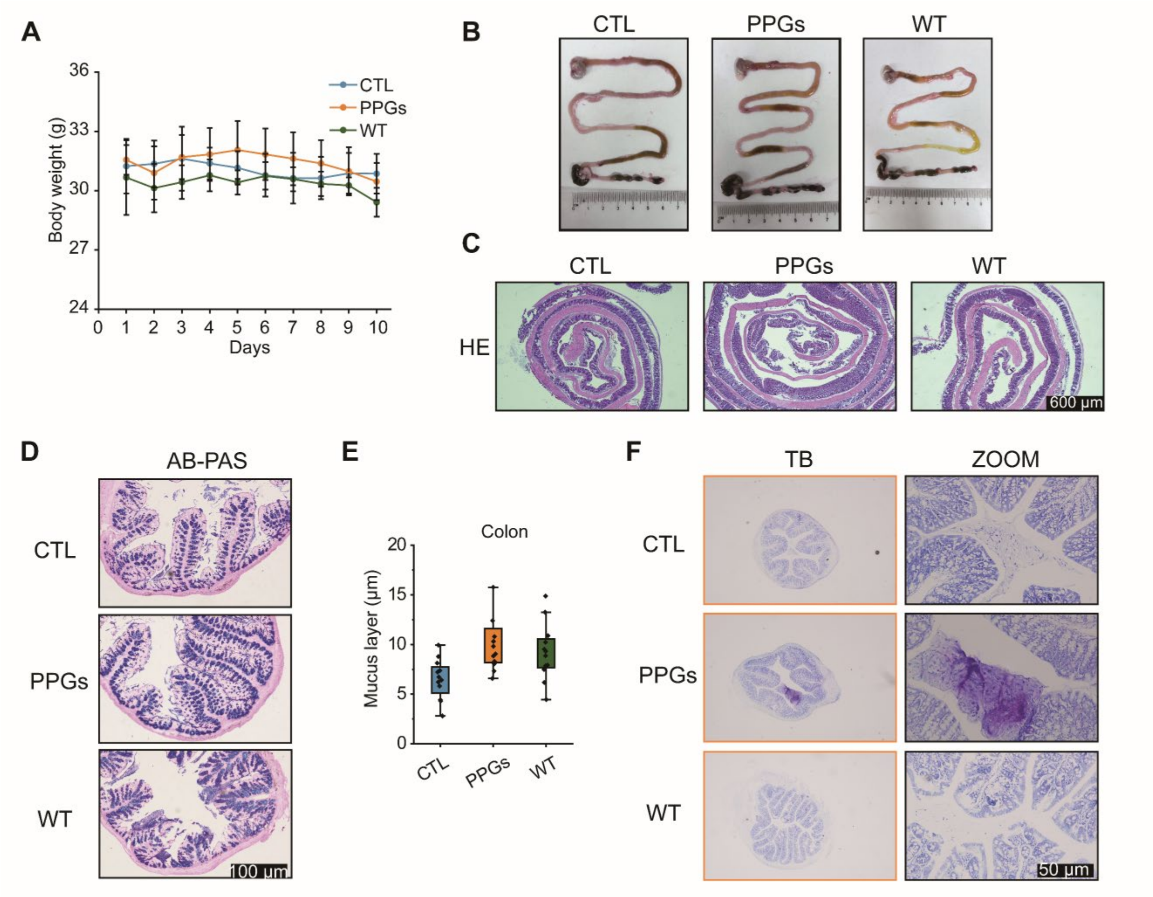


**Figure S4 Oral administration of PPGs does not induce pathological changes in the colons of healthy mice.** Healthy mice were orally gavaged for ten days, with the CTL group receiving PBS, the PPGs group receiving PPGs solution, and the WT group left untreated. (A) Body weight changes of mice during the experimental period. (B) Representative image of the full intestine. (C) Haematoxylin and eosin-stained images of the representative colon tissue. (D-E) AB–PAS staining is used to assess colonic mucus thickness, followed by statistical analysis. (F) TB staining is used to indicate the accumulation of PPGs in the colon. Data are presented as mean ± standard deviation (n = 3). The same color represents samples from the same group. $*P < 0.05$, $**P < 0.01$ by one-way ANOVA.

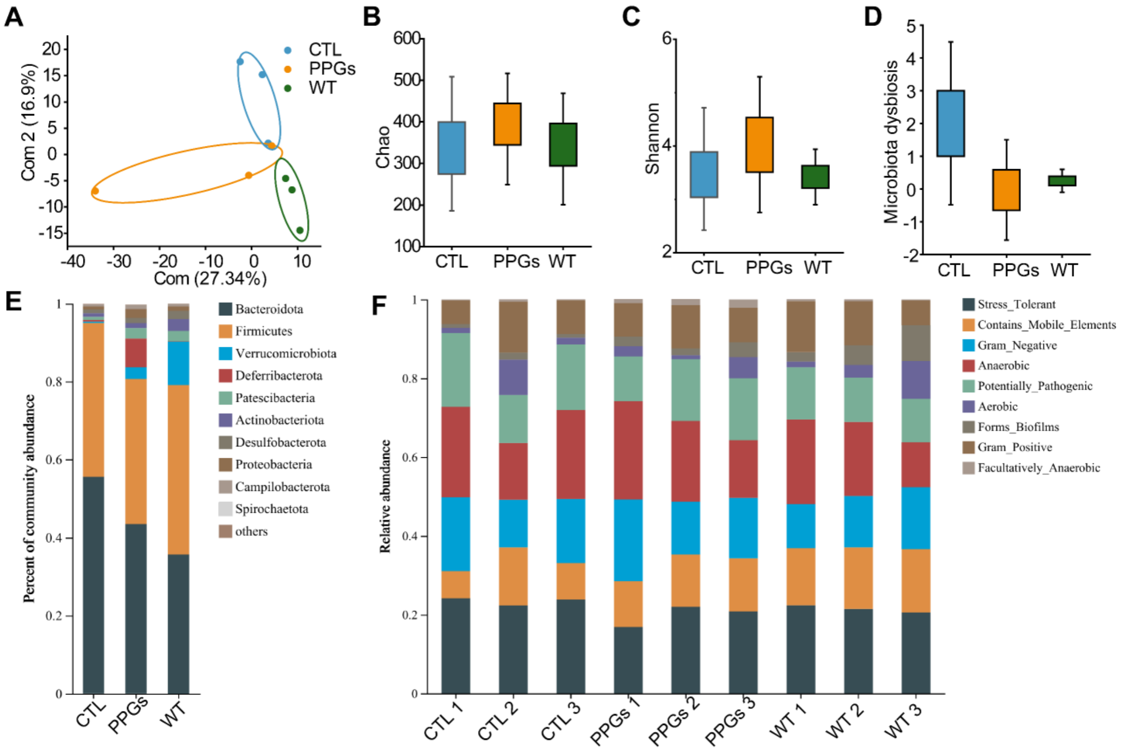


**Figure S5 Oral administration of PPGs does not alter the colonic microbiota of healthy mice.**

(A) Principal component analysis (PCA) of microbial β-diversity based on unweighted UniFrac distances, illustrating compositional differences among groups. (B-C) Alpha diversity assessed using the Chao and Shannon index, reflecting species richness and evenness. (D) Microbiota dysbiosis index, quantifying deviations from a healthy microbial profile. (E) Classify according to the Phylums. (F) Classify according to the potential functions. Data are presented as mean ± standard deviation (n = 3). The same color represents samples from the same group. *P < 0.05, **P < 0.01 by one-way ANOVA.

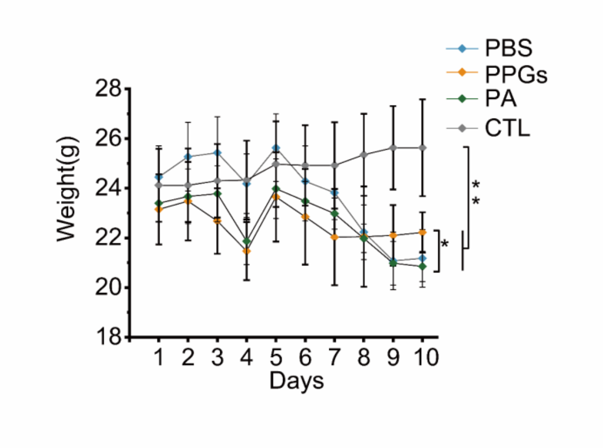


**Figure S6. Body weight changes of mice during the experimental period**

Data are presented as mean ± standard deviation (n = 6). The same color represents samples from the same group. Data are expressed as mean ± SD. *P < 0.05, **P < 0.01 by one-way ANOVA.

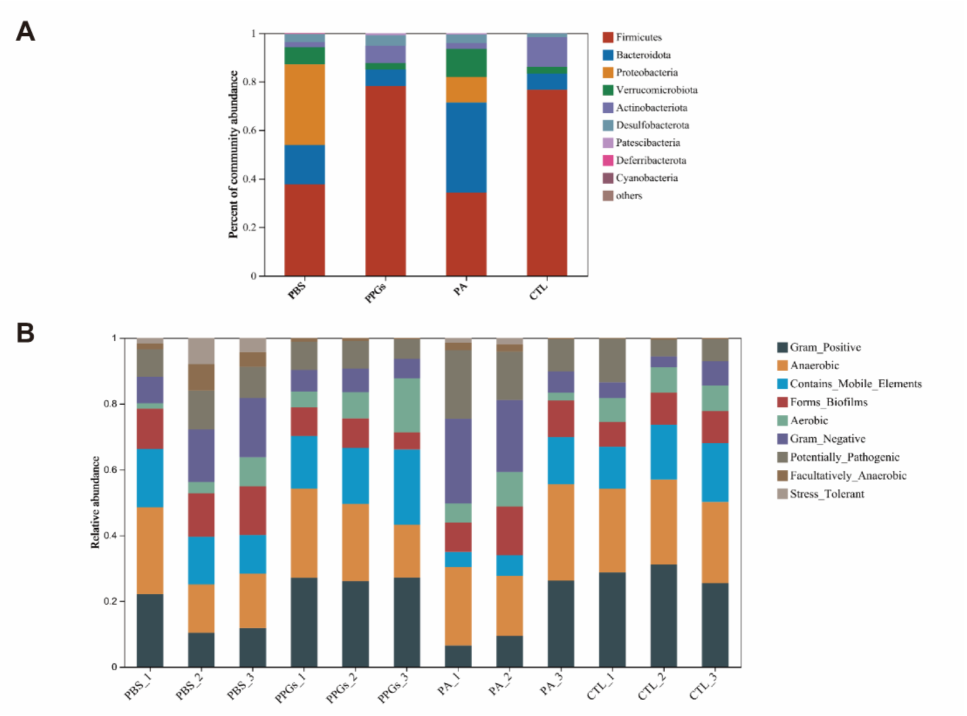


**Figure S7. Classification of gut microbiota**

(A) Classify according to the Phylums

(B) Classify according to the potential functions
